# Endoscopic ultrasound–directed transenteric endoscopic retrograde cholangiopancreatography using antegrade hepaticogastrostomy-assisted saline instillation in Roux-en-Y anatomy: a case report

**DOI:** 10.1016/j.igie.2025.11.002

**Published:** 2025-11-17

**Authors:** Timothy Adam, Michiel Bronswijk, Jan Vandewalle, Giuseppe Vanella, Enrique Pérez-Cuadrado-Robles, Roy L.J. van Wanrooij, Schalk Van der Merwe

**Affiliations:** 1 Department of Gastroenterology and Hepatology, University Hospital Gasthuisberg, University of Leuven, Leuven, Belgium; 2 Department of Gastroenterology and Hepatology, Imelda Hospital Bonheiden, Bonheiden, Belgium; 3 Vita-Salute San Raffaele University, Milan, Italy; 4 Pancreatobiliary Endoscopy and Endosonography Division, IRCCS San Raffaele Scientific Institute, Milan, Italy; 5 Department of Gastroenterology, Georges-Pompidou European Hospital, Paris, France; 6 Amsterdam Gastroenterology Endocrinology Metabolism, Amsterdam, the Netherlands; 7 Cancer Center Amsterdam, Amsterdam, the Netherlands; 8 Department of Gastroenterology and Hepatology, Amsterdam UMC, Vrije Universiteit Amsterdam, Amsterdam, the Netherlands

## Abstract

Endoscopic ultrasound (EUS)-directed transenteric endoscopic retrograde cholangiopancreatography (EDEE) has become a valuable therapeutic option in patients with nongastric bypass anatomy requiring biliary access. However, adequate afferent loop access can be challenging. Our aim was to illustrate an alternative way of achieving small-bowel distention during EDEE in a patient with recurrent cholangitis due to strictures of both the left- and right-sided bilioenteric anastomosis. We used a temporary EUS-guided hepaticogastrostomy to provide antegrade afferent loop access, facilitating on-demand saline instillation and small-bowel distention. Antegrade catheter–based saline instillation provided adequate afferent loop distention and stabilization for the creation of an enteroenteric anastomosis with a lumen-apposing metal stent (LAMS). This allowed for adequate through-the-LAMS endoscopic retrograde cholangiopancreatography and stricture management in nongastric bypass Roux-en-Y anatomy. We concluded that antegrade hepaticogastrostomy–assisted saline instillation might be useful in patients undergoing EDEE where classic approaches to afferent loop distention have failed.

## Introduction

The reported incidence of hepaticojejunostomy strictures after surgical repair of iatrogenic bile duct injury varies from 10% to 15% to upward of 30% of patients. Knowledge regarding risk factors, such as concomitant vascular injury, bile leak, and repair by a nonspecialist surgeon,^1^ as well as data on minimally invasive treatment of these anastomotic strictures, has increased considerably in the last decade. Whereas percutaneous drainage was once the sole minimally invasive option, a wide range of effective endoscopic options has become available. We describe a complex case where endoscopic ultrasound (EUS)-guided antegrade transgastric saline instillation was used, which facilitated adequate afferent loop access and further stricture management.

## Case description

A 72-year-old patient was transferred to our tertiary care department after recurring bouts of cholangitis with anorexia, fever, and jaundice despite prolonged antimicrobial prophylaxis. The medical history included a Roux-en-Y hepaticojejunostomy, with a need for a bilateral hepaticojejunostomy after complex bile duct trauma during cholecystectomy 20 years earlier. There was no relevant family or social history. Biochemical analysis revealed significantly elevated cholestatic liver test results (alanine aminotransferase 106 U/L [reference: ≤41 U/L], gamma-glutamyltransferase 836 U/L [reference: ≤60 U/L], and total bilirubin 2.9 mg/dL [reference: ≤0.5 mg/dL]). Magnetic resonance cholangiopancreatography showed progressive intrahepatic bilateral bile duct dilation and confirmation of 2 separate bilioenteric anastomoses (Fig. 1). Given the complex postsurgical history, the patient preferred a minimally invasive approach. The decision was made not to attempt enteroscopy-assisted endoscopic retrograde cholangiopancreatography (ERCP)^2^^,^^3^ but to use EUS-directed transenteric ERCP (EDEE)^4^^,^^5^ because this would facilitate simultaneous treatment of both anastomoses and allow for more straightforward endoscopic reaccess if required.

**Figure 1:**
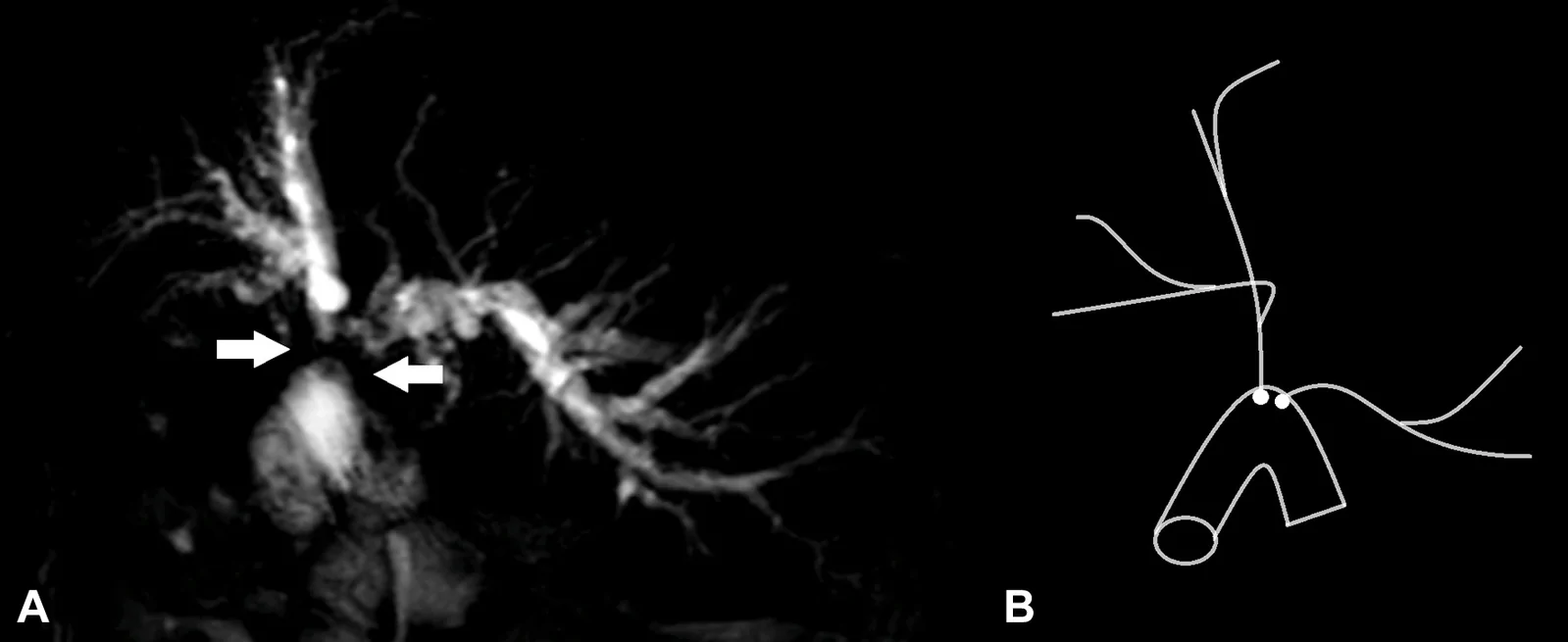
**A,** Magnetic resonance imaging showing bilateral intrahepatic bile duct dilation and tapering toward the bilioenteric anastomoses (*arrows*). **B,** Schematic overview of the 2 separate intrahepatic bilioenteric anastomoses showing independent left- and right-sided drainage.

A linear echoendoscope (EG38-J10UT; Pentax Medical, Tokyo, Japan) was introduced and confirmed left-sided bile duct dilation. After intubation of the native duodenal bulb and despite changing the position from supine to left lateral, no optimal visualization of the afferent loop was obtained. The decision was made to perform EUS-guided hepaticogastrostomy aimed at antegrade filling of the afferent loop (Video 1, available online at www.igiejournal.org). The dilated duct in segment 3 (B3) was punctured with a 19-gauge needle (EchoTip Ultra; Cook Medical, Bloomington, Ind, USA), followed by guidewire insertion and consolidation of the trajectory with a 6F cystotome (Fig. 2, *arrow*) (Cysto-Gastro-Set; Endoflex GmbH, Voerde, Germany). A fully covered self-expandable metal stent (SEMS) was subsequently placed (80 × 10 mm, WallFlex; Boston Scientific, Marlborough, Mass, USA). One week later,^6^ the hepaticogastrostomy was cannulated using a diagnostic catheter (MTW Endoskopie W. Haag KG, Wezel, Germany), and a hydrophilic guidewire (VisiGlide; Olympus Corporation, Tokyo, Japan) was manipulated across the left-sided stenotic hepaticojejunostomy into the afferent loop (Fig. 3), facilitating placement of a 7F nasobiliary catheter (Fig. 4). Saline instillation was commenced, and the ERCP scope (TJF-Q190 V; Olympus Corporation) was exchanged for the linear echoendoscope. From the native bulb, the dilated afferent loop was successfully identified, and a 20- × 10-mm lumen-apposing metal stent (LAMS) was inserted (Hot AXIOS; Boston Scientific) (Fig. 5). As single-session ERCP was pursued, LAMS fixation was attempted with an over-the-scope stent fixation device (stentFIX; Ovesco AG, Tübingen, Germany).^7^ However, the device could not pass through the upper esophageal sphincter, and therefore, the LAMS was secured using a through-the-scope clip. After we carefully transversed the LAMS, the left-sided system could be cannulated safely with a forward-viewing gastroscope (GIF-H190; Olympus Corporation). The transgastric SEMS was subsequently removed, and a temporary 10F double-pigtail plastic stent (Solus; Cook Medical) was placed through the hepaticogastrostomy trajectory across the bilioenteric anastomosis, providing adequate left-sided drainage. As the right-sided system could not be cannulated, percutaneous transhepatic bile duct drainage (PTBD) was performed 4 days later. In the same session, through-the-LAMS right-sided access was achieved with a therapeutic gastroscope (GIF-1TH190; Olympus Corporation), and 6-mm balloon dilation (Hurricane; Boston Scientific) was performed for the right-sided anastomotic stricture (Fig. 6). To improve patient comfort and reduce the number of reinterventions, the PTBD drain was exchanged for a short 10F × 5-cm straight plastic stent during the same procedure, after which the percutaneous trajectory was closed with a dedicated tract sealing device (Hunter; Teleflex, Morrisville, NC, USA). The biliary stent was successfully inserted in a retrograde fashion without any resistance due to preceding balloon dilation, resulting in complete biliary drainage (Fig. 7). All procedures were performed in the supine position, and antimicrobial prophylaxis was administered during transmural interventions. No procedure-related adverse events occurred, and bilirubin levels returned to normal. After 3 months, elective ambulatory ERCP revealed stricture regression, after which both stents were exchanged for 2 straight 10F plastic stents, whereas the LAMS was left in place to guide future endoscopic therapy. Elective LAMS exchange was planned after 12 months to prevent potential ingrowth, and the patient expressed his gratitude that surgical reintervention could be averted.^2^^,^^3^

**Figure 2:**
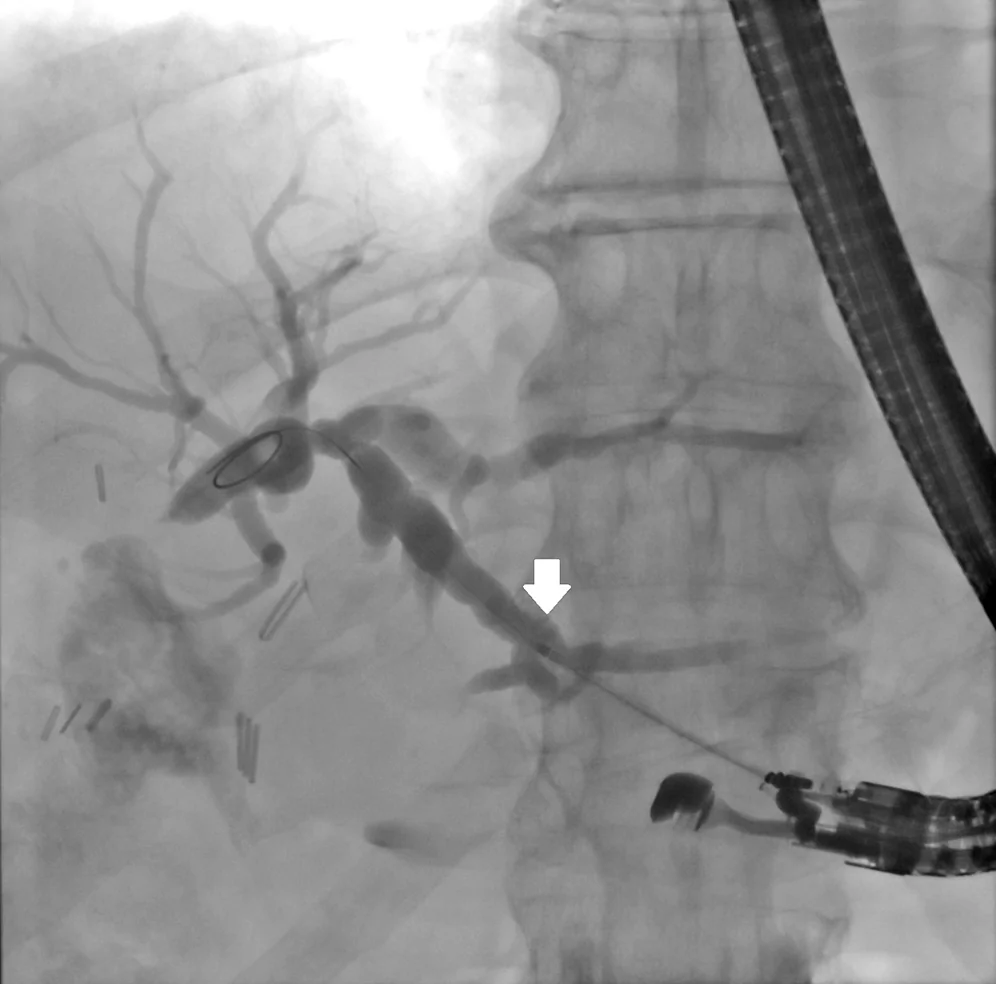
Fluoroscopy image during endoscopic ultrasound–guided hepaticogastrostomy after successful guidewire insertion and trajectory preparation using a 6F cystotome (*arrow*). Index procedure duration: 41 minutes.

**Figure 3:**
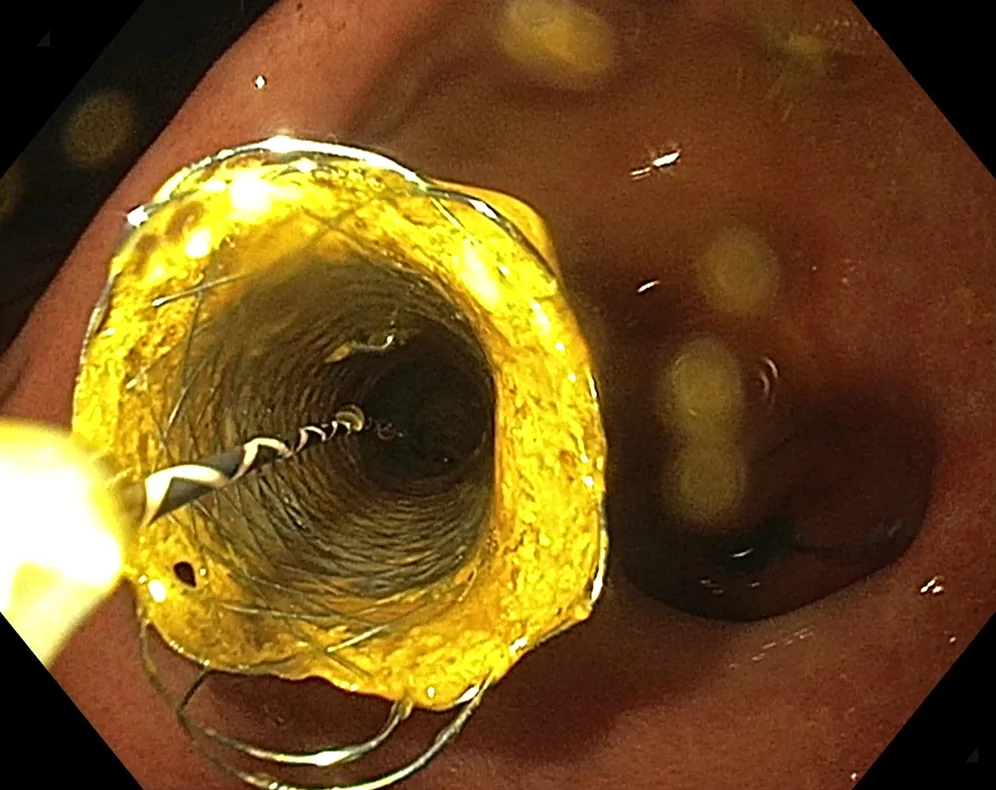
Endoscopic image during cannulation of the self-expandable metal stent after hepaticogastrostomy tract maturation.

**Figure 4:**
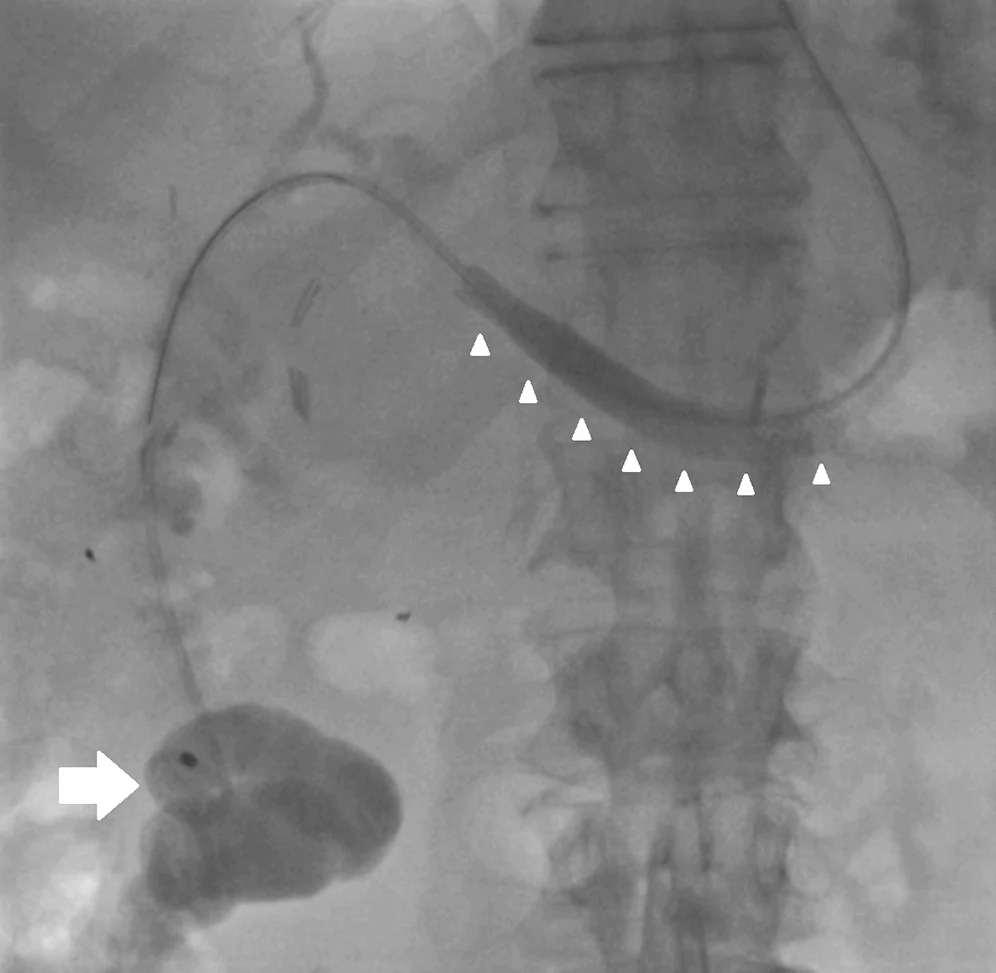
Fluoroscopy image during the same reintervention 1 week later. Successful placement of a nasobiliary catheter through the fully covered metal stent (*arrowheads*) across the hepaticojejunostomy stricture into the afferent loop (*arrow*). No signs of malignancy were seen.

**Figure 5:**
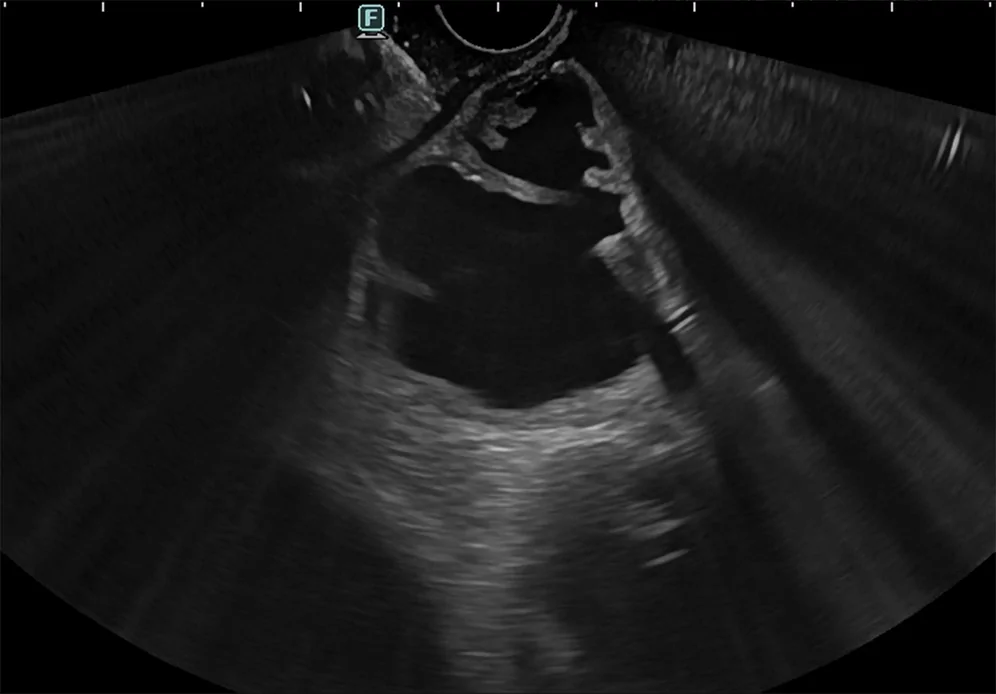
Endoscopic ultrasound image showing adequate afferent loop distention after successful antegrade catheter-assisted saline instillation. Overall procedure duration: 78 minutes.

**Figure 6:**
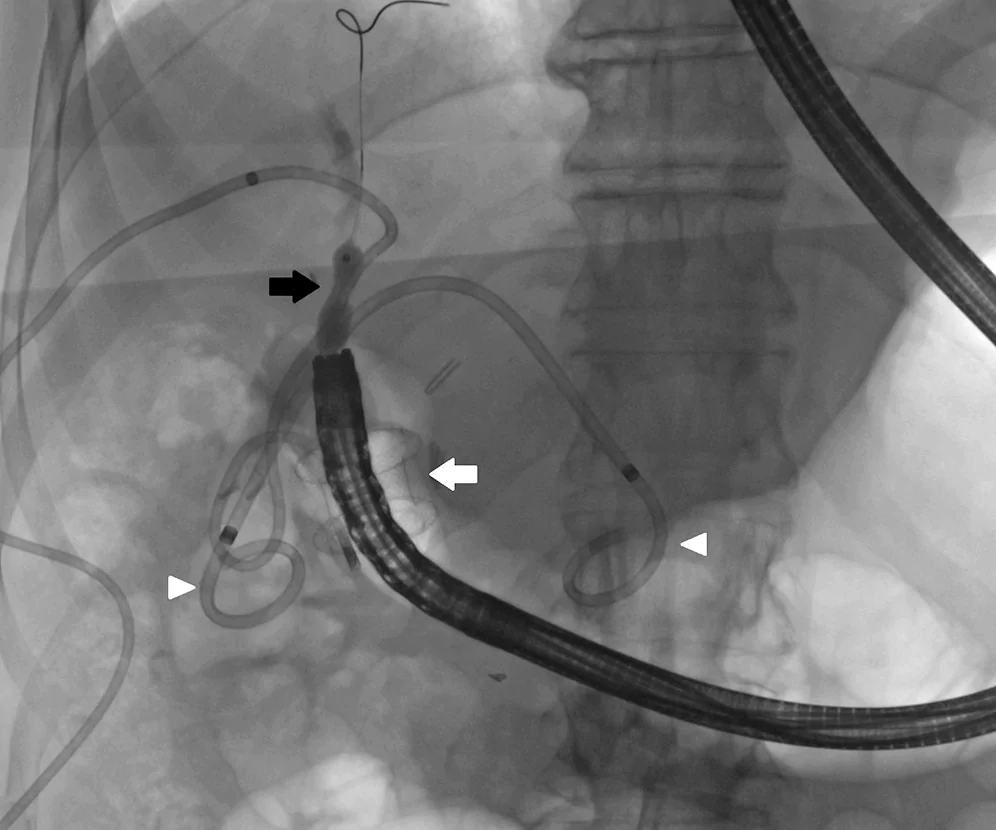
Fluoroscopy image showing successful through-the-lumen-apposing-metal-stent (*white arrow*) right-sided access and retrograde balloon dilation (*black arrow*) along the percutaneous transhepatic bile duct drain, as well as previous transgastric double-pigtail plastic stent placement (*arrowheads*) from the stomach through the left bilioenteric anastomosis.

**Figure 7:**
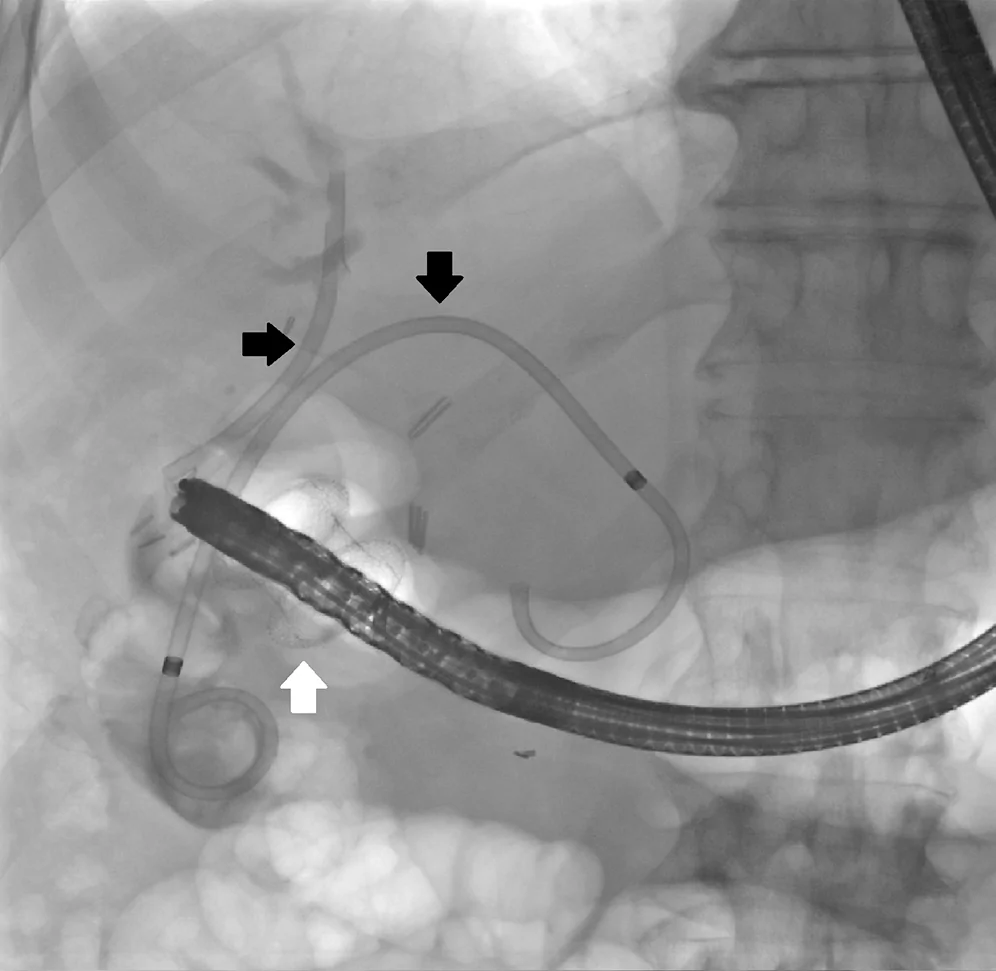
Final result with successful through-the-lumen-apposing-metal-stent (*white arrow*) bilateral 10F plastic stenting (*black arrows*).

## Discussion

Preceding surgery can present a genuine challenge to the interventional endoscopist when biliary access is required. Existing options such as enteroscopy-assisted ERCP and PTBD are plagued by either suboptimal efficacy or a high risk of adverse events, respectively. EUS-guided afferent loop access using EDEE seems to overcome these limitations by allowing for adequate endoscopic treatment, especially in patients who might require sphincterotomy or in patients with complex surgical anatomy.^4^^,^^8^ Large-caliber LAMSs furthermore support the use of scopes with large working channels, broadening the possibilities for endoscopic treatment such as digital cholangioscopy or adequate stricture management.^9^ Successful transenteric anastomosis depends heavily on adequate afferent loop identification, dilation, and stabilization. Although saline instillation by direct needle puncture, PTBD catheters,^10^ and endoscopically placed retrograde catheters have been described, the current technique involving antegrade nasobiliary catheter placement might provide a valuable EUS-based alternative for patients undergoing EDEE, allowing for continuous saline instillation in cases with challenging afferent loop access. Potential disadvantages may include the prerequisite for successful transgastric bile duct access and the need for multiple steps allowing for tract maturation, as well as mastery of each individual technique. In conclusion, we describe a patient with complex benign bilioenteric strictures that were managed successfully using EDEE via antegrade transgastric saline instillation. This particular technique might be useful in patients undergoing EDEE where classic approaches to afferent loop distention have failed.

## Patient Consent

Patient-informed consent and local Institutional Review Board approval were obtained (s71220).

## Disclosure

The following authors disclosed financial relationships: M. Bronswijk: Trial support from Boston Scientific and consultancy agreements with Ovesco Ag, Dekra, and Prion Medical. G. Vanella: Lecture fees from Boston Scientific and travel grants from Pentax Medical and Euromedical. E. P. Cuadrado-Robles: Consultant for Boston Scientific. R. L. J. van Wanrooij: Consultancy agreement with Boston Scientific. S. van der Merwe: Cook Chair in Interventional Endoscopy, consultancy agreements with Cook, Pentax, and Olympus, and co-chairs the Boston-Scientific Chair in Therapeutic Biliopancreatic Endoscopy. All other authors disclosed no financial relationships.

## Appendix

Video 1 Endoscopic ultrasound–directed transenteric endoscopic retrograde cholangiopancreatography using antegrade hepaticogastrostomy–assisted saline instillation in Roux-en-Y anatomy.
